# Prx1 Expressing Cells Are Required for Periodontal Regeneration of the Mouse Incisor

**DOI:** 10.3389/fphys.2019.00591

**Published:** 2019-05-22

**Authors:** Seyed Hossein Bassir, Sasan Garakani, Katarzyna Wilk, Zahra A. Aldawood, Jue Hou, Shu-Chi A. Yeh, Charles Sfeir, Charles P. Lin, Giuseppe Intini

**Affiliations:** ^1^Division of Periodontology, Department of Oral Medicine, Infection, and Immunity, Harvard School of Dental Medicine, Boston, MA, United States; ^2^Department of Periodontology, School of Dental Medicine, Stony Brook University, Stony Brook, NY, United States; ^3^Advanced Microscopy Program, Center for Systems Biology and Wellman Center for Photomedicine, Massachusetts General Hospital, Harvard Medical School, Boston, MA, United States; ^4^Department of Periodontics and Preventive Dentistry, University of Pittsburgh School of Dental Medicine, Pittsburgh, PA, United States; ^5^University of Pittsburgh McGowan Institute for Regenerative Medicine, Pittsburgh, PA, United States; ^6^Harvard Stem Cell Institute, Cambridge, MA, United States

**Keywords:** periodontal regeneration, periodontal stem cells, human PDLSC, Prx1, periodontal development

## Abstract

Previous studies have shown that post-natal skeletal stem cells expressing Paired-related homeobox 1 (PRX1 or PRRX1) are present in the periosteum of long bones where they contribute to post-natal bone development and regeneration. Our group also identified post-natal PRX1 expressing cells (pnPRX1+ cells) in mouse calvarial synarthroses (sutures) and showed that these cells are required for calvarial bone regeneration. Since calvarial synarthroses are similar to dentoalveolar gomphosis (periodontium) and since there is no information available on the presence or function of pnPRX1+ cells in the periodontium, the present study aimed at identifying and characterizing pnPRX1+ cells within the mouse periodontium and assess their contribution to periodontal development and regeneration. Here we demonstrated that pnPRX1+ cells are present within the periodontal ligament (PDL) of the mouse molars and of the continuously regenerating mouse incisor. By means of diphtheria toxin (DTA)-mediated conditional ablation of pnPRX1+ cells, we show that pnPRX1+ cells contribute to post-natal periodontal development of the molars and the incisor, as ablation of pnPRX1+ cells in 3-days old mice resulted in a significant enlargement of the PDL space after 18 days. The contribution of pnPRX1+ cells to periodontal regeneration was assessed by developing a novel non-critical size periodontal defect model. Outcomes showed that DTA-mediated post-natal ablation of pnPRX1+ cells results in lack of regeneration in periodontal non-critical size defects in the regeneration competent mouse incisors. Importantly, gene expression analysis of these cells shows a profile typical of quiescent cells, while gene expression analysis of human samples of periodontal stem cells (PDLSC) confirmed that Prx1 is highly expressed in human periodontium. In conclusion, pnPRX1+ cells are present within the continuously regenerating PDL of the mouse incisor, and at such location they contribute to post-natal periodontal development and regeneration. Since this study further reports the presence of PRX1 expressing cells within human periodontal ligament, we suggest that studying the mouse periodontal pnPRX1+ cells may provide significant information for the development of novel and more effective periodontal regenerative therapies in humans.

## Introduction

Periodontal diseases are characterized by bacterial-induced chronic inflammation that causes destruction of tooth supporting structures, including periodontal ligament (PDL), cementum, and alveolar bone (Pihlstrom et al., 2005). The goal of periodontal treatment is to stop the progression of the disease and regenerate the structure and function of the damaged tissues. However, a complete regeneration of these damaged structures is still unpredictable, especially in challenging clinical situations (Giannobile, 2014; Reynolds et al., 2015).

Adult skeletal stem cells may represent an effective therapeutic tool for periodontal regeneration due to their plasticity and ability to differentiate into different mesodermal cell lineages (Intini, 2010; Bassir et al., 2016). The current stem cell-based regenerative treatment modalities are based on *ex vivo* expansion of stem cells and their transplantation into the body. However, there are major practical limitations in the clinical applicability of the stem cell transplantation approaches (Prockop, 2009; Chen et al., 2011). Hence, an emerging philosophy relies on the development of treatment modalities that harness regenerative potential of endogenous stem cells *in situ* (Dimmeler et al., 2014; Zhou and van den Beucken, 2016).

The human body has the capacity to regenerate and repair through stem cells residing in the different tissues even without external therapeutic intervention (Chen et al., 2011). It is well known that stem cell niches are present in many adult tissues including PDL (Seo et al., 2004; Morrison and Spradling, 2008), and stem cells may remain in the quiescence state in their niches until they are activated in response to a regenerative need (Scadden, 2006; Morrison and Spradling, 2008). Activated stem cells may exit the niche and proliferate, self-renew, and differentiate to regenerate lost structures (Chen et al., 2011). Thus, harnessing, *in situ*, the regenerative potential of the endogenous stem cells has gained increasing attention as a simpler, safer, and more applicable alternative to stem cell transplantation, especially for the regeneration of tissues with low regenerative capacity, such as cardiac tissue, neural tissue, and tendons (Mohapel and Brundin, 2004; Ranganath et al., 2012; Lee et al., 2015). Therefore, discovering the endogenous stem cells that contribute to repair and regeneration in each tissue and understanding the molecular mechanisms that control these stem cells may lead to development of novel regenerative approaches.

Post-natal cells expressing the paired-related homeobox protein 1 (PRX1 or PRRX1) (pnPRX1+ cells), a transcription factor expressed in the mesenchyme during craniofacial and limb development (Martin and Olson, 2000; Logan et al., 2002), have been shown to have characteristics of skeletal stem cells (Cserjesi et al., 1992; ten Berge et al., 1998; Lu et al., 1999; Kawanami et al., 2009; Lu et al., 2011; Du et al., 2013; Ouyang et al., 2014; Wilk et al., 2017). More recent studies have also shown that pnPRX1+ cells of the periosteum of long bones have a significant role in post-natal development (Moore et al., 2018) and post-natal fracture repair (Duchamp de Lageneste et al., 2018). Importantly, we have recently demonstrated that pnPRX1+ cells are present in calvarial synarthroses (sutures) of adult mice and that they are required for bone regeneration of calvarial bone defects (Wilk et al., 2017). Since dentoalveolar gomphosis (periodontium), due to the limited range of motion, are similar to calvarial synarthroses, and since there is no information available on the presence or function of pnPRX1+ cells in the periodontium, the present study aimed at identifying and characterizing pnPRX1+ cells within the mouse periodontium and assess their contribution to periodontal development and regeneration.

Investigating the presence and function of pnPRX1+ cells in the periodontium may provide valuable information for understanding the periodontal regenerative mechanisms and for harnessing the endogenous regenerative potential of periodontal stem cells. Here we hypothesize that pnPRX1+ cells exist in the mouse periodontium and that in such place they represent a population of cells involved in post-natal development and regeneration.

## Materials and Methods

### Animals

All protocols and procedures were approved by the Institutional Animal Care and Use Committee (IACUC) at the Harvard University. The transgenic mouse lines utilized in this study include: (1) Prx1-creER-EGFP (MGI ID 4355342, available at Jackson Laboratory with Sock No. 029211) and (2) Prx1-creER-EGFP;Rosa26-DTA, obtained by crossing the Prx1-creER-EGFP mice with Rosa 26-DTA mice (MGI ID 3829362, available at Jackson Laboratory with Stock No. 009669).

### Distribution of Post-natal PRX1 Expressing Cells in the Mouse Periodontium

Multiphoton intravital microscopy (IVM) (Celso et al., 2009) and Prx1-creER-EGFP transgenic mice (Kawanami et al., 2009) were utilized to explore the presence and distribution in the mouse periodontium of cells co-expressing the Prx1 gene and green fluorescent protein (GFP) (*n* = 3). Briefly, fresh mandible bones were harvested from 3- and 8-week old male Prx1-creER-EGFP mice at the experiment date. The tissue was embedded in OCT compound and transferred into liquid nitrogen. Embedded samples were trimmed on a cryostat to get a flat surface for imaging. The samples were sectioned with 20 μm step-size until the periodontal ligament was fully detectable in each section. The laser wavelength was tuned to 900 nm and focused into the sample through a 60× water objective (numerical aperture = 1). The laser power was kept constant at 30 mW on the sample. The fluorescent signal from prx1-eGFP+ cells and second harmonic generation (SHG) signal from collagen fibers in the bone were collected in the epi direction. The signals were separated with a 485 nm dichroic mirror and detected by photomultiplier tubes with corresponding optical filters (465 nm short pass filter for SHG signal and 525/50 nm bandpass filter for eGFP sginal). The samples were imaged on a 3D stage manually to scan through the periodontal ligament. For each field of view, a stack of 60 images were taken with 2 μm step-size. The images were processed and analyzed in ImageJ software (US National Institutes of Health, United States).

### Inducible Lineage Ablation Study and Post-natal Periodontal Development

To generate a Prx1-creER-EGFP;Rosa26-DTA mouse line (Ablation mouse line) we crossed male Prx1-creER-EGFP mice with mice engineered to conditionally express diphtheria toxin A (DTA) upon cre recombination of a loxP-flanked STOP sequence (Rosa26-DTA mice) (Voehringer et al., 2008). This system allows for the *in vivo* cell-specific activation of the diphtheria toxin A. In this mouse line, DTA is expressed only conditionally, upon induction of cre recombination by tamoxifen. Upon injection of tamoxifen, all cells expressing PRX1 start expressing DTA, which leads to apoptosis and global ablation. Efficacy of ablation of PRX1+ cell was previously assessed in calvarial tissue (80–90% ablation efficiency) (Wilk et al., 2017) and re-assessed in mandibular tissues (90–100% ablation efficiency) (**Appendix Figure 1**).

The following mouse groups were used: (1) the test group consisted of Prx1-creER-EGFP^+/-^;Rosa26-DTA^+/-^. In these mice, ablation of the pnPRX1+ cells occurs after treatment with tamoxifen due to the co-presence of the creER-EGFP and the DTA transgenes; (2) the control group consisted of littermates of the test group mice: either Prx1-creER-EGFP^+/-^;Rosa26-DTA^-/-^ or Prx1-creER-EGFP^-/-^;Rosa26-DTA^+/-^. In these mice, ablation of the pnPRX1+ cells does not occur – even upon treatment with tamoxifen – because the creER-EGFP and the DTA transgenes are not co-expressed. In both test and control groups, animals (*n* = 5) at post-natal age of 3 days were treated with tamoxifen (Intraperitoneal, 40 mg/kg in sterile oil), daily for 10 consecutive days (Wilk et al., 2017). Animals were sacrificed at the post-natal age of 21 days. Mandibles were collected and fixed in 70% ethanol. All samples underwent micro-CT scanning (SCANCO μCT 35, Scanco Medical, Bruttisellen, Switzerland; source voltage: 70 kVp, power: 8 W, exposure time: 300 ms, and voxel size: 10 micron). Width of periodontal ligament spaces was measured in the right mandibular incisor and in the mesial and distal roots of right mandibular first molar. Measurements were taken using Avizo software package (version 9.2.0 FEI Visualization Sciences Group, MA, United States) based on methodologies described elsewhere (Niver et al., 2011).

### Preparation of Non-critical Size Periodontal Fenestration Defects

**FIGURE 1:**
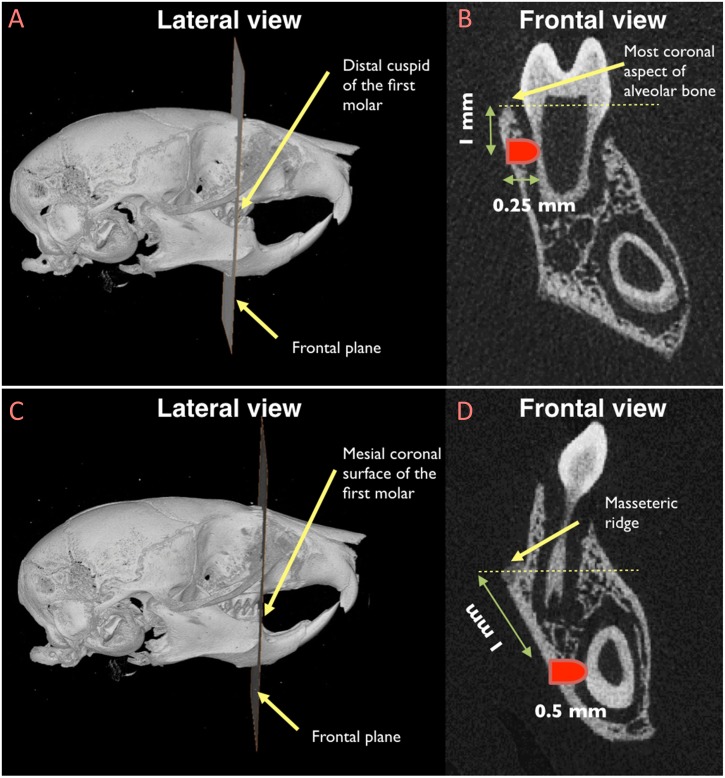
Location of the sub-critical size fenestration periodontal defects in the mandibular first molar **(A and B)** and in the mandibular incisor **(C and D)**. **(A)** The distal cuspid of the first molar was used as the reference point to locate the frontal plane in correspondence of which the periodontal defect by the mandibular first molar would be created; **(B)** once the reference frontal plane was identified, the exact location of the defect was recognized by measuring a distance of 1 mm from the most coronal aspect of the alveolar bone; **(C)** the mesiocoronal surface of the first molar was utilized as the reference point to locate the frontal plane in correspondence of which the periodontal defect by the mandibular incisor would be created; **(D)** after identifying the reference frontal plane, the periodontal defect was created 1 mm apical to the masseteric ridge.

To create non-critical size periodontal defects, an extra-oral incision was made at the lower border of the mouse mandible. After separating superficial fascia, master muscle, and periosteum, the alveolar bone was exposed. The molar defect was generated as follow: the distal cuspid of the first molar was considered as the reference point and the defect was created 1 mm below the most coronal aspect of alveolar bone at the level of distal cuspid (Figure 1). Dental burs were utilized, by hand, to create a defect with 0.5 mm in diameter, penetrating into the bone for 0.25 mm, thus creating a defect in the alveolar bone, in the PDL, and in the dentin of the distal root of the first molar. The incisor defect was generated using the mesiocoronal surface of the first molar as the reference point (Figure 1). The defect was created 1 mm apical to the masseteric ridge at the level of mesiocoronal surface of the first molar. The incisor defect was 0.5 mm in diameter and 0.5 mm in depth to involve the alveolar bone, the PDL, and the dentin of the mandibular incisor. Defects were left unfilled to allow spontaneous healing. Then, soft tissue were repositioned and the extra-oral incision was sutured using continuous suturing technique with absorbable 5-0 Vicryl sutures (Hu-Friedy, Chicago, IL, United States). Following surgery, animals were given injection of Buprenorphine (subcutaneous injection, 0.05 mg/kg) for pain control at the time of surgery and every 12 h for the first 48 h.

### Inducible Lineage Ablation Study and Periodontal Regeneration

To test the effects of global ablation of pnPRX1+ cells on the regeneration of non-critical size periodontal defects, we compared the healing of the defects between a test group (ablated PRX1 expressing cells) and the control group (non-ablated PRX1 expressing cells).

The test group mice (Prx1-creER-EGFP^+/-^;Rosa26-DTA^+/-^ mice) and the control group littermates (Prx1-creER-EGFP^+/-^; Rosa26-DTA^-/-^ or Prx1-creER-EGFP^-/-^;Rosa26-DTA^+/-^ mice) consisted of 7- to 9-week old male mice (*n* = 5). In each mouse, two periodontal fenestration non-critical size defects were created by the right mandibular incisor and the first mandibular molar teeth and all mice were treated with tamoxifen (Intraperitoneal, 40 mg/kg in sterile oil) for 5 days preoperatively and 5 days post-operatively (Wilk et al., 2017). After an eight-week healing period, animals were sacrificed. Mandibles were collected and fixed in 70% ethanol and Micro-CT and histological analyses were performed.

### Evaluation of Healing of the Periodontal Fenestration Defects

Micro-CT and histological analyses were performed to assess the healing of non-critical size periodontal fenestration defects.

Micro-CT analysis: All samples underwent micro-CT scanning (SCANCO μCT 35, Scanco Medical; source voltage: 70 kVp, power: 8 W, exposure time: 300 ms, and voxel size: 10 micron). The healing of periodontal defects was assessed using Avizo software package (version 9.2.0 FEI Visualization Sciences Group, MA, United States), and the healing was reported as (1) completely healed, (2) partially healed, or (3) not healed. The defects were considered “completely healed” when a bony bridge formed across the defect borders and osseous continuity of mandible was restored. The defects were scored as “partially healed” if a bony bridge was not completely formed in all sections. When no bony bridge formed inside the defect, it was considered as “not healed.”

Histological analysis: Samples were prepared for cryosectioning and histological analysis after micro-CT scanning. Sections were stained with Hematoxylin and Eosin (H&E) and prepared for light microscopy.

### Gene Expression Study of Mouse PRX1 Expressing Cells

The gene expression profile of PRX1 expressing cells isolated from PDL of mouse mandibular incisors was investigated. Prx1-creER-EGFP^+/-^ transgenic mice were used (*n* = 7). In this mouse model, all cells expressing PRX1 co-express green fluorescent protein. 4-week old male animals were sacrificed, mandibular incisors were extracted, and PDL tissues were separated from the root surface of the teeth. The obtained PDL tissues were pooled and digested at 37°C in a shaking water bath in two steps to prepare single-cell suspensions. GFP+ (expressing PRX1) and GFP- (not expressing PRX1) cells were sorted and isolated by means of Fluorescent-Activated Cell Sorting (FACS). Cell lysis, RNA isolation, and reverse transcription for gene expression analysis were performed using a Single Cell to CT Kit (Ambion by Life Technologies, Carlsbad, CA, United States) according to manufacturer’s protocols. Quantitative reverse transcription polymerase chain reactions (qRT-PCR) were carried out on RNA isolated from pulled cells obtained from 7 animals/group, with three technical replicates for each gene evaluation. Comparative threshold cycle method was used to analyze the data (Schmittgen and Livak, 2008).

We investigated and compared the expression of the genes in the following categories between GFP+ and GFP- cells: (A) progenitor cell and stem cell markers: Cd44, Pdgfra, Smo, and Col2a1; (B) Wnt signaling pathway genes: Axin2, Wif1; (C) Notch signaling pathway genes: Dll4, Jag1, Notch1, and Notch2; and (D) genes related to cell-cycle regulatory markers, adhesion molecules, and other relevant genes: Mcm4, Itgb1, Foxo3, and Pth1r. GADPH served as endogenous reference gene.

The following TaqMan^^®^^ assays (Applied Biosystems, ThermoFisher Scientific, Inc., United States) were utilized for the mouse quantitative gene expression analysis: Mm01277163_m1 for CD44; Mm00440701_m1 for Pdgfra; Mm01162710_m1 for Smo; Mm01309565_m1 for Col2a1; Mm00443610_m1 for Axin2; Mm00442355_m1 for Wif1; Mm00444619_m1 for Dll4; Mm00496902_m1 for Jag1; Mm00627185_m1 for Notch1; Mm00803077_m1 for Notch2; Mm00725863_s1 for Mcm4; Mm01253230_m1 for Itgb1; Mm01185722_m1 for Foxo3; Mm00441046_m1 for Pth1r; and Mm99999915_g1 for GAPDH.

### Gene Expression Study of Human PDLSC

We quantified transcript levels of Scleraxis, MCAM/CD146, and Prx1 (transcriptional variant 1 and 2) in total RNA obtained from human primary PDLSCs (kindly provided by Dr. Pamela Robey, NIH/NIDCR – samples collected under IRB-approved protocol at NIH, #06-D-0144) (2 biological replicates, 2 technical replicates for each gene evaluation), and from primary Human Dermal Microvascular Endothelial Cells (HDMEC, PromoCell Inc., United States) (2 biological replicates, 2 technical replicates for each gene evaluation). Total RNA was isolated using miRNeasy kit (Qiagen, Valencia, CA, United States) according to the manufacturer’s instructions. First-strand cDNAs were synthesized using Transcriptor First Strand cDNA Synthesis Kit (Roche Diagnostics, Indianapolis, IN, United States) according to the manufacturer’s protocol. Real-time PCR was performed on an Applied Biosystems StepOnePlus Real-Time PCR system (Applied Biosystems, Foster City, CA, United States). Tubb5 and ACTB served as endogenous reference genes and geometrical average of their *C*t values was utilized for the comparative threshold cycle analysis, as described in detail elsewhere (Schmittgen and Livak, 2008).

The following TaqMan^^®^^ assays (Applied Biosystems, ThermoFisher Scientific, Inc., United States) were utilized for the human quantitative gene expression analysis: Hs03054634_g1 for Scleraxis (Scx); Hs00174838_m1 for MCAM; Hs01014477_m1 for Prx1 variant 1; Hs01025547_m1 for Prx1 variant 2; Hs00742828_s1 for Tubb5; and Hs01060665_g1 for ACTB.

### Statistical Analysis

Student’s *t*-test was used to compare the width of PDL space between groups. Prior to performing the statistical evaluation, a power analysis was performed to determine the final sample size with a 95% confidence interval (α = 0.05). The width of periodontal space was considered as the primary outcome variable and the analysis of the preliminary data indicated a sample size of *n* = 4 to be sufficient to provide 80% power to recognize a significant difference of 0.02 mm (and standard deviation of 0.01 mm) between groups. One additional animal was included in each group to account for possible unexpected mortality (sample size on = 5).

Student’s *t*-test was used to compare the relative levels of expression for each gene between pnPRX1+ cells and pnPRX1- cells and the Δct values of genes expressed in PDLSC and HDMEC.

The healing of the periodontal defects was compared using Fisher’s exact test. A significance level of *a* = 0.05 was used for all comparisons.

## Results

### PnPRX1+ Cells Are Present Within PDL of Mouse Incisor

**FIGURE 2:**
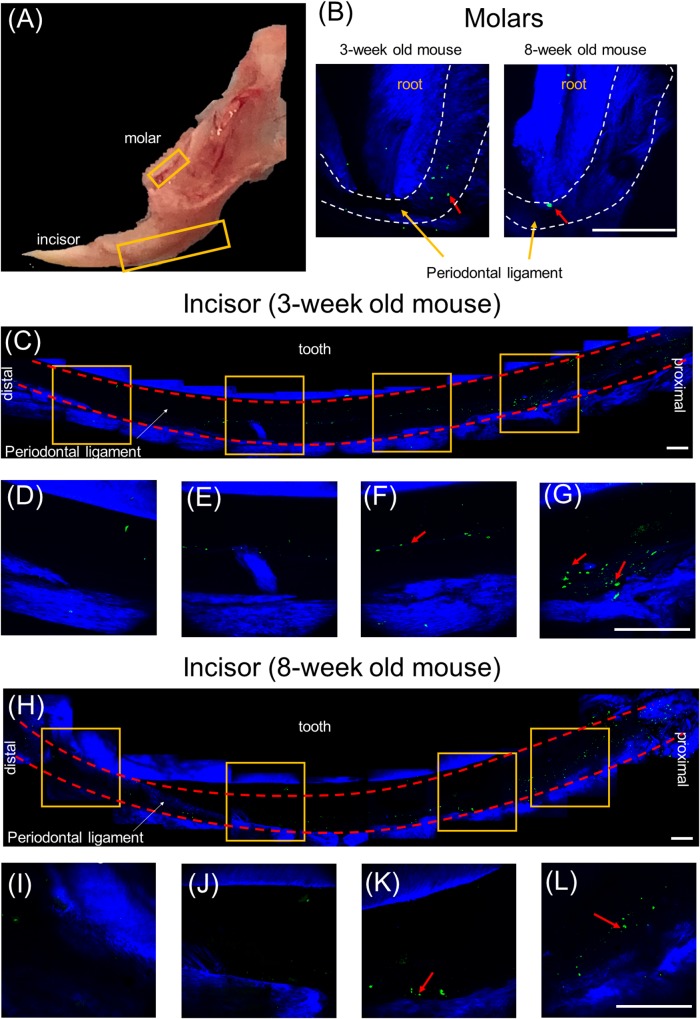
Multiphoton intravital microscopy images of pnPRX1+ cells in mice periodontal ligaments. The pnPRX1+ cells (green) express GFP and the bones and tooth (blue) were visualized by their SHG signals. **(A)** Fresh harvested mouse mandible bone; **(B)** multiphoton images of periodontal ligaments of a 3-week old and 8-week old mouse molars. Dashed white lines demark PDL space boundary; **(C)** multiphoton images of lower periodontal ligaments of a 3-week old mouse incisor; **(D–G)** zoomed-in images of the lower mouse incisor periodontal ligament corresponding to the labeled gold squares in **(C)**; **(H)** multiphoton images of lower periodontal ligaments of a 8-week old mouse incisor; **(I–L)** zoomed-in images of the lower mouse incisor periodontal ligament corresponding to the labeled gold squares in **(H)**. The scale bars are 200 μm. The red arrows point at pnPRX1+ cells.

Our previous study, based on histological slides of short pulsing lineage tracing of Prx1creER-EGFP;tdTOMATO mice, indicated that pnPRX1+ cells are located in the tissue surrounding the incisor (Wilk et al., 2017). To test whether any pnPRX1+ cells, although in lower number, may be present in proximity of the mouse molars as well, here we utilized high resolution IVM analysis and investigated the presence of GFP expressing cells in the mandible of Prx1creER-EGFP^+/-^ mice (Figure 2A). Our evaluation reveals the presence of pnPRX1+ cells in the PDL of both molars (Figure 2B) and incisors (Figure 2C–L) in 3- and 8-week old mice. Although not quantitative, this IVM-based high resolution evaluation confirmed that pnPRX1+ cells are present in the PDL of the mandibular incisors (as previously reported) (Wilk et al., 2017) and revealed that fewer pnPRX1+ cells are present in the PDL of the mandibular molars. It further appears that while pnPRX1+ cells can be consistently found in the PDL of the incisor at both tested ages (Figure 2C–L), they seem to decrease in number over time in the molar’s PDL (Figure 2B).

### PnPRX1+ Cells Contribute to Post-natal Periodontal Development

To test whether pnPRX1+ cells of the molar and the incisor have a significant role on postnatal development of their periodontium, we genetically ablated pnPRX1+ cells by means of tamoxifen induced expression of DTA in 3-day old mice and tested the effect of such ablation on the width of the periodontium after 18 days, in 3-week old mice.

**FIGURE 3:**
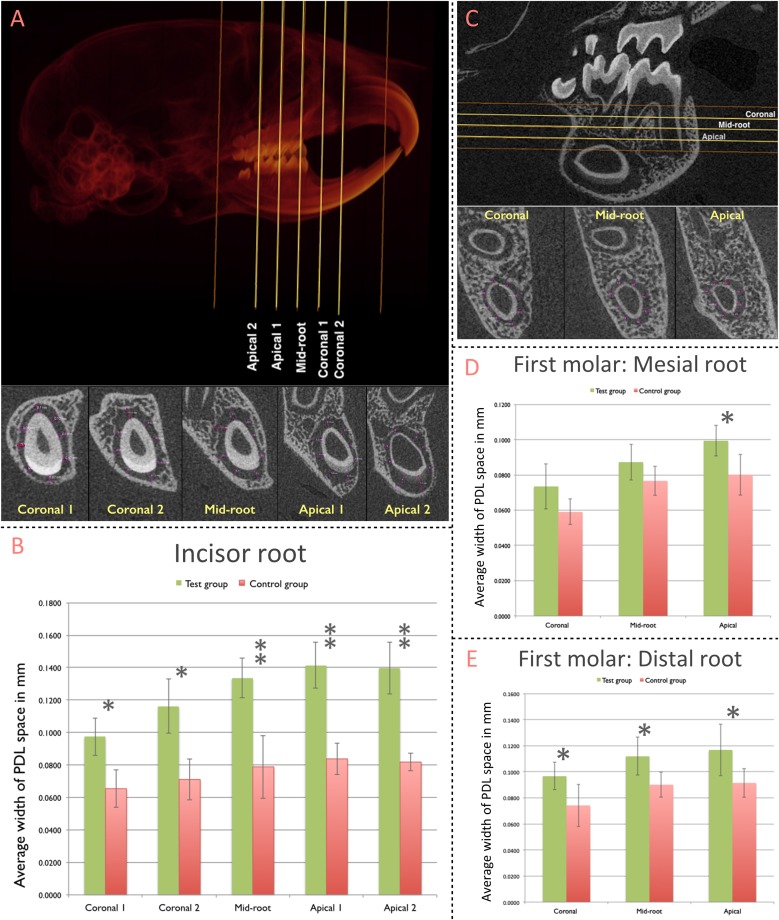
Contribution of pnPRX1+ cells to the post-natal development of the molar and incisor periodontium. **(A)** The width of PDL space around the root of the right mandibular incisor was measured in five transverse slices (Coronal 1, Coronal 2, Mid-root, Apical 1, and Apical 2 slices; top image). These transverse slices were generated based on the two indicated reference points (orange lines): the most coronal level of the alveolar bone (coronal reference) and the most apical point of the tooth socket (apical reference). The linear measurements of the width of PDL space were performed at 8 different points within each section (bottom image, purple arrows). The measurements at the enamel surface (buccal measurement in the most of slices) were excluded from the analysis. All 8 linear measurements were averaged and such average was considered as the width of the PDL space for each section. **(B)** Comparison of the width of PDL space of the mandibular incisor tooth between the test and the control groups. **(C)** The width of the PDL space around mesial root of right mandibular first molars was measured in three transverse slices (Coronal, Mid-root, and Apical - yellow lines) generated based on two reference points (orange lines): the furcation entrance of the tooth (coronal reference) and the apex of the mesial root (apical reference). Similar methodology was used to select the slices for the distal root of the first molars. The linear measurement was performed at 8 different points, similarly, to what was performed for the incisor tooth. **(D)** Comparison of the width of PDL space of mesial root of the mandibular first molar; and **(E)** distal root of the mandibular first molar between test and control groups. (^∗^*p* < 0.05, ^∗∗^*p* < 0.001).

The Width of the PDL space around the mandibular incisor was assessed at 5 standardized sections along the root length (Figure 3A). Compared to the control group (non-ablation group), width of PDL space was significantly greater in the test group (ablation group) for all five sections (Coronal 1: *p* = 0.002; Coronal 2: *p* = 0.001; Mid-root: *p* < 0.001; Apical 1: *p* < 0.001; Apical 2: *p* < 0.001) (Figure 3B).

The width of PDL space around mesial and distal roots of the mandibular first molar was measured at three standardized sections along the root length (Figure 3C). The difference in the width of PDL space around the mesial root was significantly greater in the test group (ablation group) compared to the control group (non-ablation group) at the Apical section (*p* = 0.017; Figure 3D). The average width of the PDL space measured among each section of the distal root was significantly greater in the test group compared to the control group for all three sections [Coronal (*p* = 0.032), Mid-root (*p* = 0.022), and Apical (*p* = 0.038)] (Figure 3E).

### PnPRX1+ Cells Are Required for Periodontal Regeneration of the Continuously Regenerating Mouse Incisor

To test whether pnPRX1+ cells of the molar and the incisor have a significant role on postnatal regeneration of their periodontium, we first determined that 0.5 mm (diameter) periodontal fenestration defects (incisors and molars) would regenerate spontaneously in wild type mice (data not shown). Thus, in the present studies we used two 0.5 mm non-critical size periodontal fenestration defects, one at the buccal aspect of the right mandibular incisor and one at the buccal surface of the distal root of the right first mandibular molar (Figure 1, 4A,G). We genetically ablated pnPRX1+ cells by means of tamoxifen induced expression of DTA in 7–9 week-old male mice and tested the effect on the regeneration of a sub-critical periodontal defect eight weeks after surgical creation of the defects.

**FIGURE 4:**
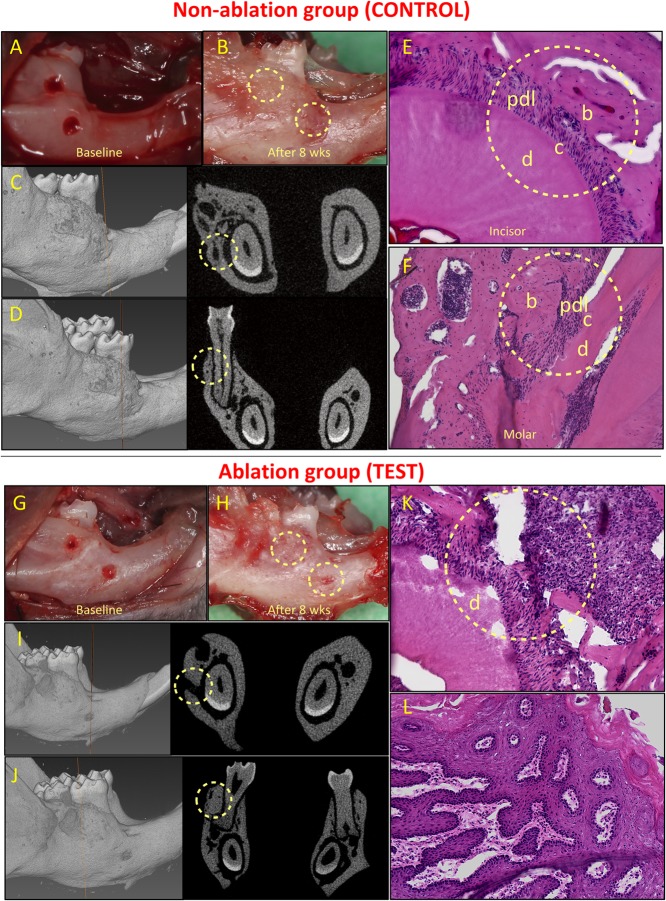
Healing of non-critical defects in non-ablation (images above the red line; A–F) and ablation (images below the red line; G–L) groups (*n* = 5). Two defects were created around the mandibular incisor and first molar teeth in both groups **(A and G)**. In the non-ablation group, healing of both defects was observed clinically for all animals **(B)**. Micro-CT analysis confirmed the healing of incisor **(C)** and molar **(D)** defects, and histological analysis confirmed regeneration of the periodontal incisor **(E)** and molar **(F)** defects. In the ablation group, lack of healing was observed for incisor defects in all animals **(H)**. Micro-CT **(I)** and histological **(K)** analyses confirmed lack of periodontal regeneration for incisor defects in this group. The molar defects were mainly healed by excessive and irregular bone formation **(H, J, L)**. b, bone; c, cementum; d, dentine; and pdl, periodontal ligament.

All animals in both groups survived after the surgeries. In the control group (non-ablation group), non-critical periodontal fenestration defects around both incisor and molar teeth healed (Figure 4A–F). Healing was observed clinically and was confirmed by means of micro-CT analysis (Figure 4B–D). Periodontal regeneration was also observed in histological analysis (Figure 4E,F). Complete healing of the incisor defects was observed in 4 out 5 animals, while the defect in one animal had partial healing (**Appendix Figure 2**). The molar defects in all five animals healed completely, but the healing was accompanied by excessive and irregular bone formation in two out of five animals (**Appendix Figure 3**).

In the test group, we found that all defects by the incisor tooth did not heal, while the defects by the molar tooth, at least in part, healed (Figure 4G–L). Specifically, for the incisor defects we observed lack of healing in the 5 out of 5 animals (**Appendix Figure 4**). For the molar defects, we observed partial healing in two cases, and complete healing in the other three defects. However, the healing was almost always accompanied by irregular and excessive bone formation (in four out of five cases, see **Appendix Figure 5**).

Fisher’s exact test showed a statistical significant difference between the test and control groups in the healing of the incisor defects (*p* = 0.008). There was no significant difference between the two groups in the healing of the molar defects (*p* = 0.44) (Table 1).

**Table 1:** Distribution of the type of healing of the defects in the test and control groups.

	Incisor defects				Molar defects			
	No healing	Partial healing	Complete healing	*p-*value^#^	No healing	Partial healing	Complete healing	*p-*value^#^
Test group	5	0	0	0.008^∗^	0	2	3	0.44
Control group	0	1	4		0	0	5	

*A significant difference was observed between test and control groups in the healing of incisor defects. ^#^Fisher’s exact test. ^∗^Significant differences between the groups.*

### PnPRX1+ Cells Express Stemness Markers

To test whether pnPRX1+ cells of the incisor present with a gene expression profile compatible with that observed in other skeletal stem cells, we FAC-sorted GFP expressing cells (GFP+ cells co-expressing PRX1) and GFP- cells from the PDL of mandibular incisors of 4-week old male PRX1-creER-EGFP^+/-^ mice.

**FIGURE 5:**
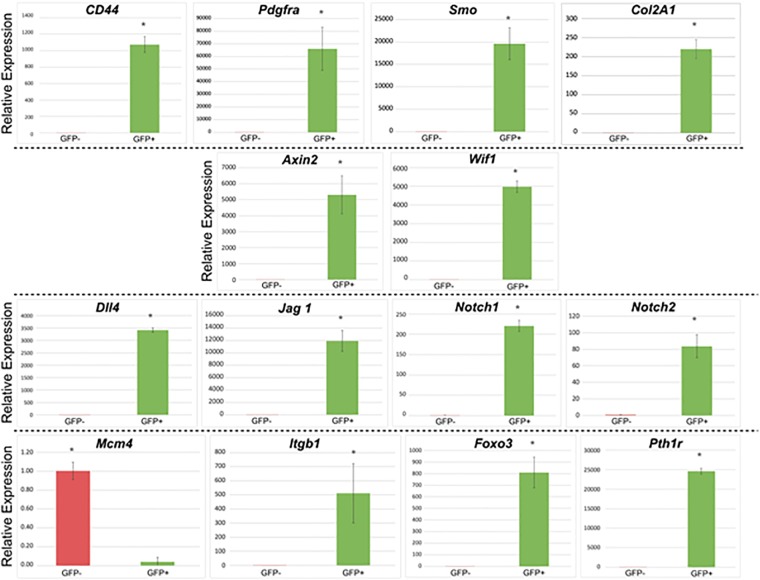
Gene expression profile of GFP+ (PRX1+) and GFP– (PRX1–) cells. Cells were isolated from the periodontal ligament of mouse mandibular incisors of 4-week-old Prx1-creER-EGFP+/- mice. ^∗^*p* < 0.05.

Results of gene expression studies are reported in Figure 5. Compared to pnPRX1- cells, pnPRX1+ cells express higher levels of markers of mesenchymal progenitor/stem cells, such as such Cd44, Pdgfra, Smo, and Col2A1 (Trzaska et al., 2007; Itzkovitz et al., 2012; Chan et al., 2015). In pnPRX1+ cells, we also observed a high level of expression of inhibitors of the Wnt signaling pathway, such as Axin2 and WNT Inhibitory Factor 1 (Wif1), frequently observed in bone quiescent cells (Kim et al., 2007; Ling et al., 2009). High levels of expression of genes encoding Notch ligands (Dll4, and Jagged-1) and Notch receptors (Notch 1 and Notch 2) were found in these cells. PnPRX1+ cells also express low levels of Mcm4, a characteristic of quiescent cells since Mcm4 expression is associated with DNA replication (Renault et al., 2009). High levels of expression of Itgb1 and Foxo3, commonly associated with stem cell homing and anchoring to the extracellular matrix of the niches (Renault et al., 2009; Cheung and Rando, 2013), were observed in pnPRX1+ cells. Finally, it was found that Pth1r was expressed at extremely higher levels in the pnPRX1+ cells compared to the pnPRX1- cells.

### Prx1 Is Expressed in Human PDLSC

**FIGURE 6:**
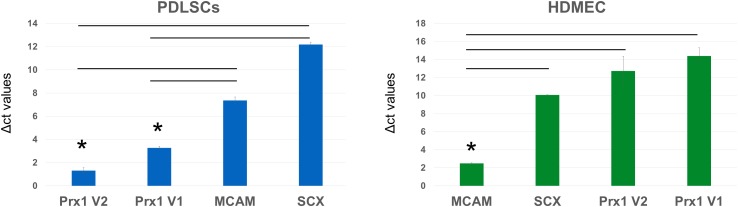
Analysis of Δct values of stem cell markers in PDLSC and HDMEC. Comparison of Δct values of MCAM/CD146, Scleraxis, and PRX1 in human PDLSC and in human HDMEC. ^∗^*p* < 0.05.

Gene expression analysis of human samples of PDLSC and HDMEC (Figure 6) revealed that in PDLSC, transcript levels of Prx1 (both transcriptional variants) are higher than transcript levels of MCAM and SCX (as indicated by lower levels of Δct values). Conversely, in HDMEC, transcripts of MCAM are higher than transcript levels of Prx1 variants (Figure 6). Considering that MCAM and SCX are two well described markers of periodontal stem cells (Seo et al., 2004; Zhu and Liang, 2015), expression of Prx1 may also be considered representative of the multi-potent human PDLSC.

## Discussion

The challenge with harnessing endogenous regenerative potential of mesenchymal stem cells for tissue regeneration is related to the low abundance of these cells in the post-natal human tissues (Orford and Scadden, 2008; Rooker et al., 2010). With the ultimate goal of defining therapeutically efficient methods to harness endogenous stem cells, we explored and studied the presence and the regenerative role of a sub-population of progenitor cells of the periodontium. This work reports the presence of PRX1 expressing cells within the mouse and the human PDL and studied the biological role of these cells in a newly developed periodontal regeneration *in vivo* mouse model.

We found that pnPRX1 expressing cells mainly reside in the PDL of the mouse incisor. They are also present, although in lower abundance, in the in the PDL of the mouse molars. These findings are somehow different from the reports of our previous studies, where histological examinations of pnPRX1+ cells in the mouse mandible (based on a short pulse induced expression of a fluorescent reporter in pnPRX1+ cells) revealed the presence of pnPRX1+ cells only in proximity of the mouse incisor (Wilk et al., 2017). Here a higher resolution analysis conducted by means of IVM reveals the presence of pnPRX1+ cells in both the incisor’s and the molar’s PDL. The mouse incisors, unlike the molars, erupt continuously throughout the life of the animal (Harada et al., 2002). Since this continuous eruption requires continuous remodeling of the PDL (Marks and Schroeder, 1996; Rooker et al., 2010), the high abundance of PRX1 expressing cells in the PDL of the mouse incisor suggests that these cells may have key roles in the continuous remodeling and regeneration of the incisor’s periodontal ligament. Although we did not perform a quantitative comparison between incisors and molars, the apparent lower abundance of pnPRX1+ cells in the PDL of the mouse molars may indicate a role in their post-natal development and may be representative of their inactive regeneration status. In fact, this study demonstrated that the width of PDL increased in both incisor and molar teeth after the ablation of PRX1 expressing cells during post-natal development, indicating that pnPRX1+ cells have an active role in the development of the width of periodontal space, a significant indicator of periodontal homeostasis (McCulloch et al., 2000; Lim et al., 2014), in both molars and incisors. Significantly, this study also found that post-natal PRX1 expressing cells are required for regeneration of non-critical periodontal defects in the continuously regenerating incisors of skeletally mature mice (7- to 9-week old mice). In fact, upon ablation of pnPRX1+ cells in these mice, in the incisors we observed lack of regeneration of non-critical periodontal defects, whereas in the molars we observed that the healing was accompanied by excessive and irregular bone formation in 4 out of 5 cases. The observed differences might be attributed to the lower abundance of pnPRX1+ cells observed in the molar’s PDL versus incisor’s PDL, which may due to a drop at the end of developmental phase.

We speculate that the excessive and irregular bone formation observed in the molars upon ablation of pnPRX1+ cells may be due to the remaining pool of differentiated osteo-competent cells (remaining PRX1 progeny, such as pre-osteoblasts and osteoblasts) that persevere with the healing process. Given their lack of multipotency, progeny cells might not be able to sustain a physiological tissue regeneration. In addition, while we were able to quantitatively evaluate bone regeneration/number of healed defects (bone bridging of the defects), we could not quantify the regeneration of the periodontal ligament itself. However, our qualitative histological evaluations seem to indicate that, in absence of pnPRX1+ cells, periodontal ligament regeneration in the molar defects occurs with irregular formation of periodontal fibers, signifying that tissue regeneration competent cells may be required for a physiological regeneration of the periodontal ligament as well.

Mice and lager animals have been largely utilized to study periodontal infection (Kantarci et al., 2015). However, animal models useful to evaluate periodontal regeneration, in absence of a periodontal infection, have only been developed in rats and other larger animals (Pellegrini et al., 2009). Thus, a significant barrier prevented mouse genetics to be utilized in periodontal regeneration studies. To overcome this barrier, we generated a new periodontal defect mouse model that allowed us to understand the role that post-natal PRX1 expressing cells have in the continuous regeneration of the mouse incisors. While this model provides valuable information about the contribution to periodontal regeneration of a specific cell population, it should also be noted that, as indicated by the abnormal bone regeneration that occurs in proximity of the created periodontal defect, the bone defect is quite severe as well. Yet, our proposed model may represent an invaluable tool to investigate the regulatory mechanisms and molecular signals that mediate self-renewal, proliferation, and differentiation of periodontal stem cells.

The presented gene expression analysis of the mouse pnPRX1+ cells indicate that these cells express a set of genes typically expressed in other stem cells. It would have been informative to test whether such gene expression signature is maintained in pnPRX1+ cells during the regeneration process. If so, we could conclude that pnPRX1+ cells maintain their stemness qualities in loco, whereas it is their osteo-competent progeny that is responsible for the actual regeneration process. Unfortunately, such analysis is limited by the inability to isolate a sufficient number of pnPRX1+ cells from the small 0.5 mm regenerating defect. Future studies using novel technologies that allow for single cell resolution (Nowotschin et al., 2019) may help answering this important question.

Similar to what has recently been done for the identification of the human skeletal stem cells (Chan et al., 2018), future studies should aim at fully charactering the mouse pnPRX1+ cells with their specific set of membrane receptors, so that the corresponding human homologs with the same set of receptors may also be identified. Understanding the regulatory mechanisms that govern the regeneration potentials of the mouse incisors’ pnPRX1+ cells and testing whether such mechanisms are present or can otherwise be stimulated in the human homologous cells, may allow for the development of future therapeutic approaches in periodontology.

In conclusion, post-natal PRX1 expressing cells are present with a biologically significant abundance within the PDL of the mouse molars and the continuously regenerating PDL of the mouse incisor. Importantly, the presence of post-natal PRX1 expressing cells within human periodontal ligament is revealed in this study. Hence, this project represents an initial step towards future investigations aimed at identifying novel and more effective regenerative therapies for the human periodontium.

## Data Availablity

All datasets generated for this study are included in the manuscript and/or the Supplementary Files.

## Ethics Statement

The part of the present study that utilized animals followed protocols and procedures approved by the Institutional Animal Care and Use Committee (IACUC) at the Harvard University. The part of the present study that utilized human samples was carried out in accordance with the recommendations of the Institutional Review Board (IRB) at NIH, with written informed consent from all subjects. All subjects gave written informed consent in accordance with the Declaration of Helsinki (NIH IRB approved protocol #06-D-0144).

## Author Contributions

SB contributed to conception and design, to data acquisition, analysis, and interpretation, drafted manuscript, critically revised manuscript, gave final approval. SG, KW, ZA, JH, and S-CY contributed to conception, contributed to data acquisition and analysis, gave final approval. CS contributed to interpretation, critically revised manuscript, gave final approval. CL contributed to conception, contributed to interpretation, critically revised manuscript, gave final approval. GI contributed to conception and design, data analysis and interpretation, drafted manuscript, critically revised manuscript, gave final approval.

## Conflict of Interest Statement

The authors declare that the research was conducted in the absence of any commercial or financial relationships that could be construed as a potential conflict of interest.
