# Supplementary material for: Prx1 Expressing Cells Are Required for Periodontal Regeneration of the Mouse Incisor
Source: Front Physiol. 2019 May 22;10:591. doi: 10.3389/fphys.2019.00591 (PMC6558369; doi:10.3389/fphys.2019.00591)
Supplement: Supplementary file 1 [file Data_Sheet_1.docx]

**Appendix**

**Prx1 Expressing Cells are Required for Periodontal Regeneration of the Mouse Incisor.**

**Authors**

Seyed Hossein Bassir, Sasan Garakani, Katarzyna Wilk, Zahra A. Aldawood, Jue Hou, Shu-Chi A. Yeh, Charles Sfeir, Charles P. Lin, Giuseppe Intini

**Materials and Methods**

**Ablation efficacy Experiment**

Adult Prx1-creER-EGFP^+/-^;DTA^-/-^ (n=3) and Prx1-creER-EGFP^+/-^;DTA^+/-^ (n=2) male mice were treated with tamoxifen (Intraperitoneal, 40mg/kg in sterile oil) for 10 consecutive days. After treatment, mandibular incisors were extracted, PDL tissues were separated from the root surface of teeth, and cells were isolated. The presence of EGFP+ cells was analyzed by FACS. Percentage of GFP+ cells/viable cells ± SD are reported for each group (Appendix Figure 1).


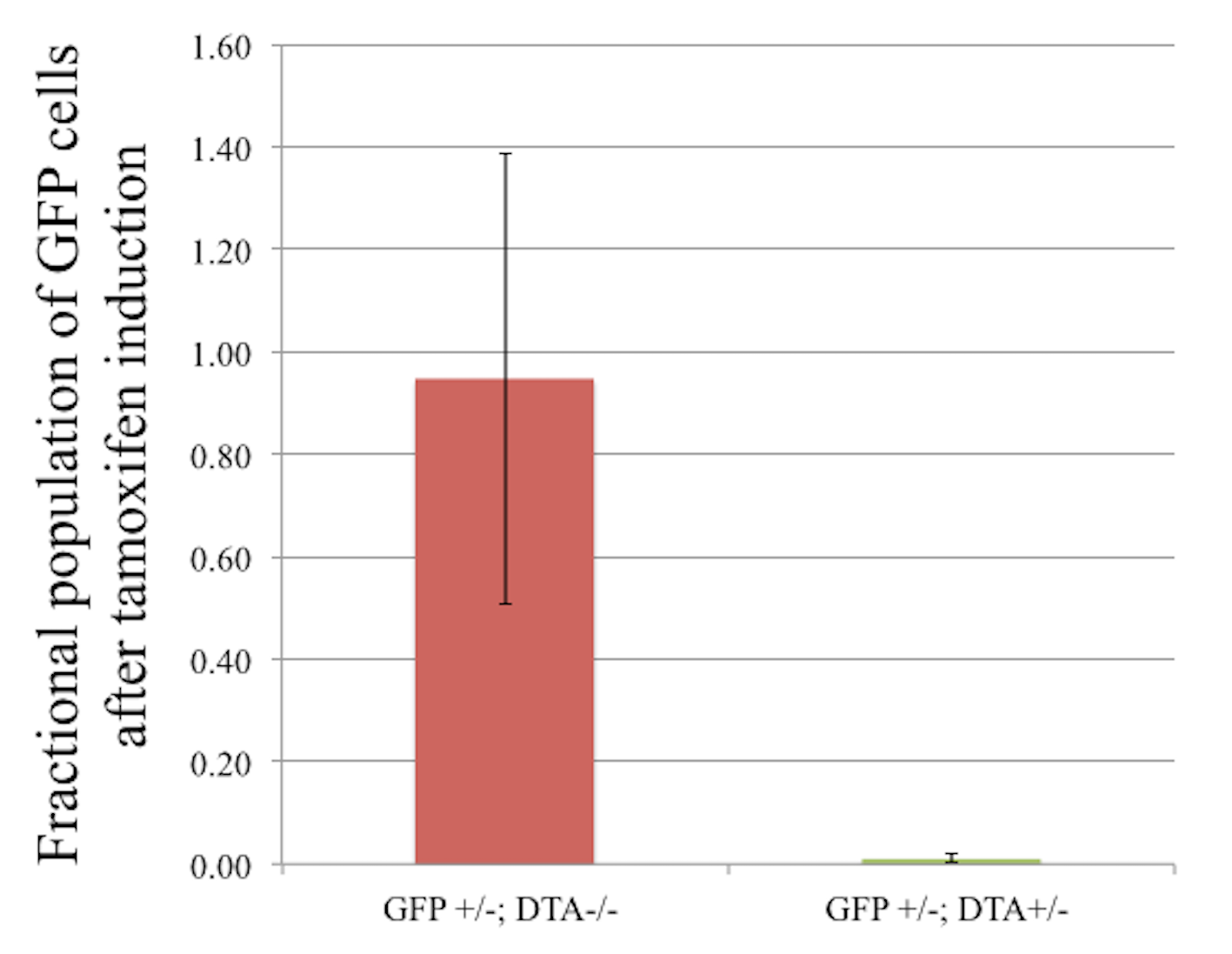


**Appendix Figure 1- Ablation efficiency of the DTA-mediated global ablation of pnPRX1+ cells.** Adult Prx1-creER-EGFP+/-;DTA-/- (3 mice) and Prx1-creER-EGFP+/-;DTA+/- (2 mice) male mice were treated with tamoxifen (Intraperitoneal, 40mg/kg in sterile oil) for 10 consecutive days. After treatment, mandibular incisors were extracted, PDL tissues were separated from the root surface of teeth, and cells were isolated. The presence of GFP+ cells was analyzed by FACS. Percentage of GFP+ cells/viable cells ± SD are reported for each group. 10 days of tamoxifen treatment resulted in an average of 98.92% reduction in the number of pnPRX1+ cells.


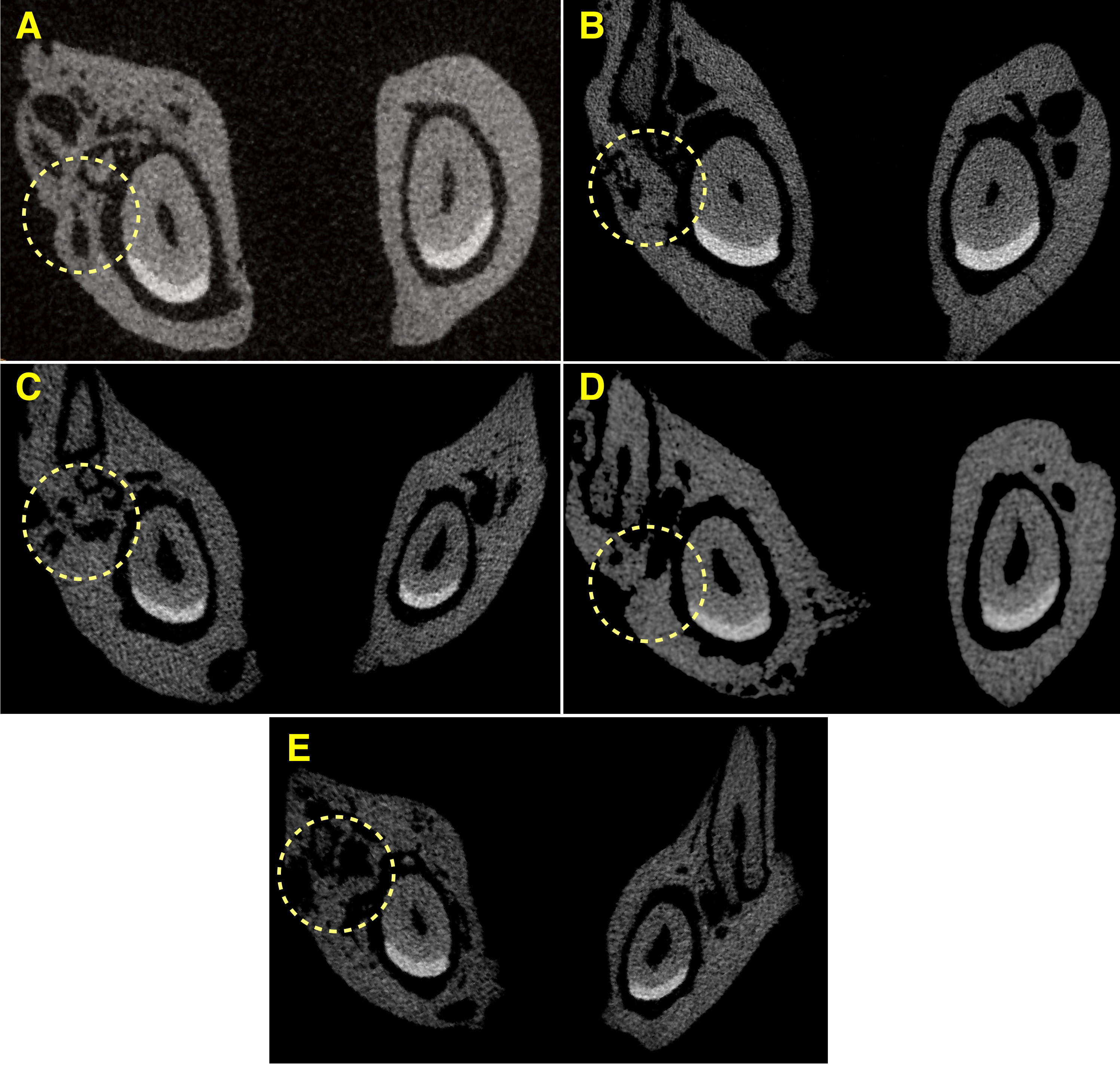


**Appendix Figure 2- Healing of non-critical defects by the incisor tooth in the control (non-ablation) group (frontal sections).** Complete healing was observed in 4 animals (A-D), and partial healing was noted in one animal (E) (n=5).

**
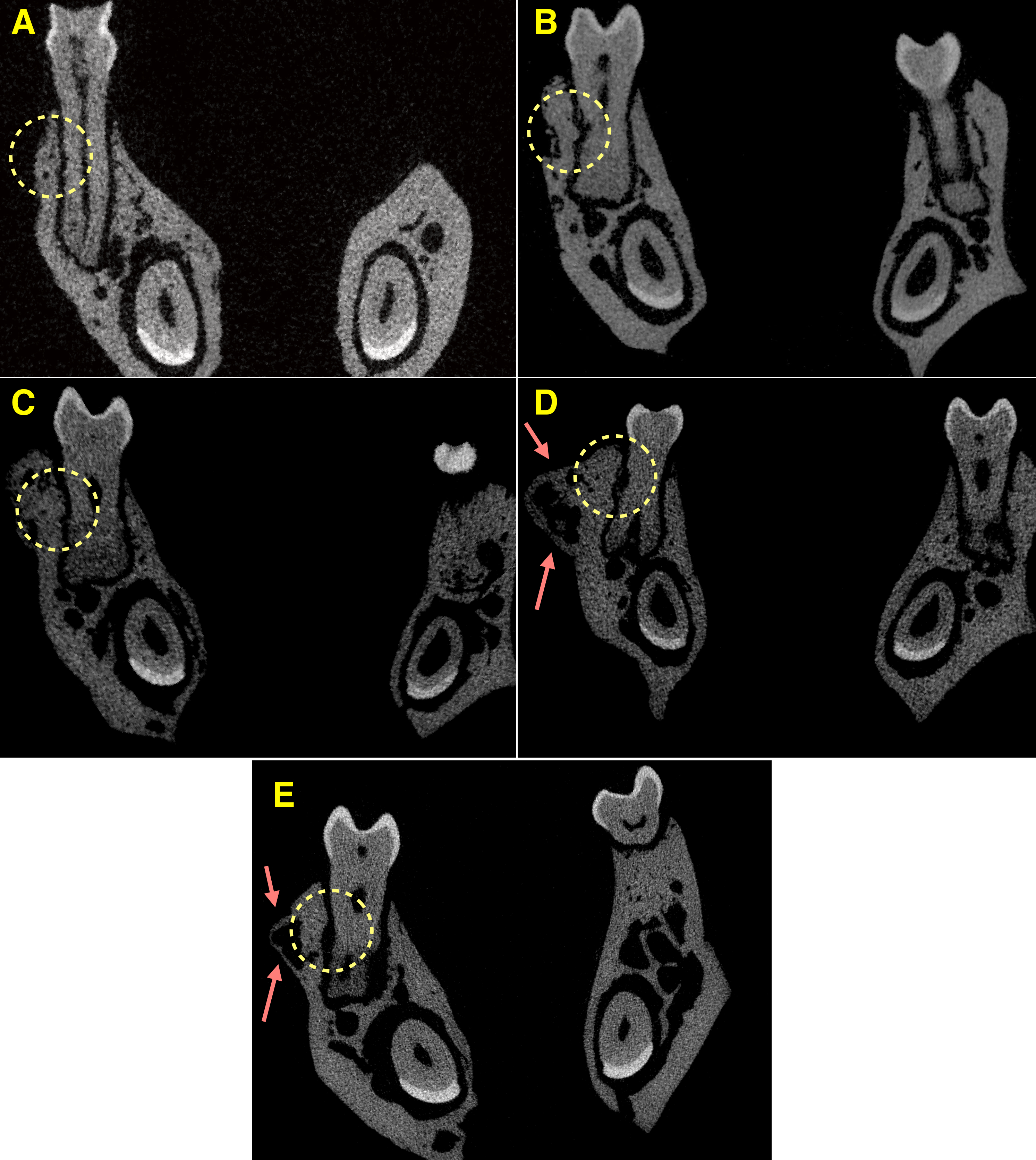
**

**Appendix Figure 3- Healing of non-critical defects by the molar tooth in the control (non-ablation) group (frontal sections).** Complete healing was observed in all defects (A-E); healing was accompanied by excessive bone formation in two animals (D and E; red arrows) (n=5).


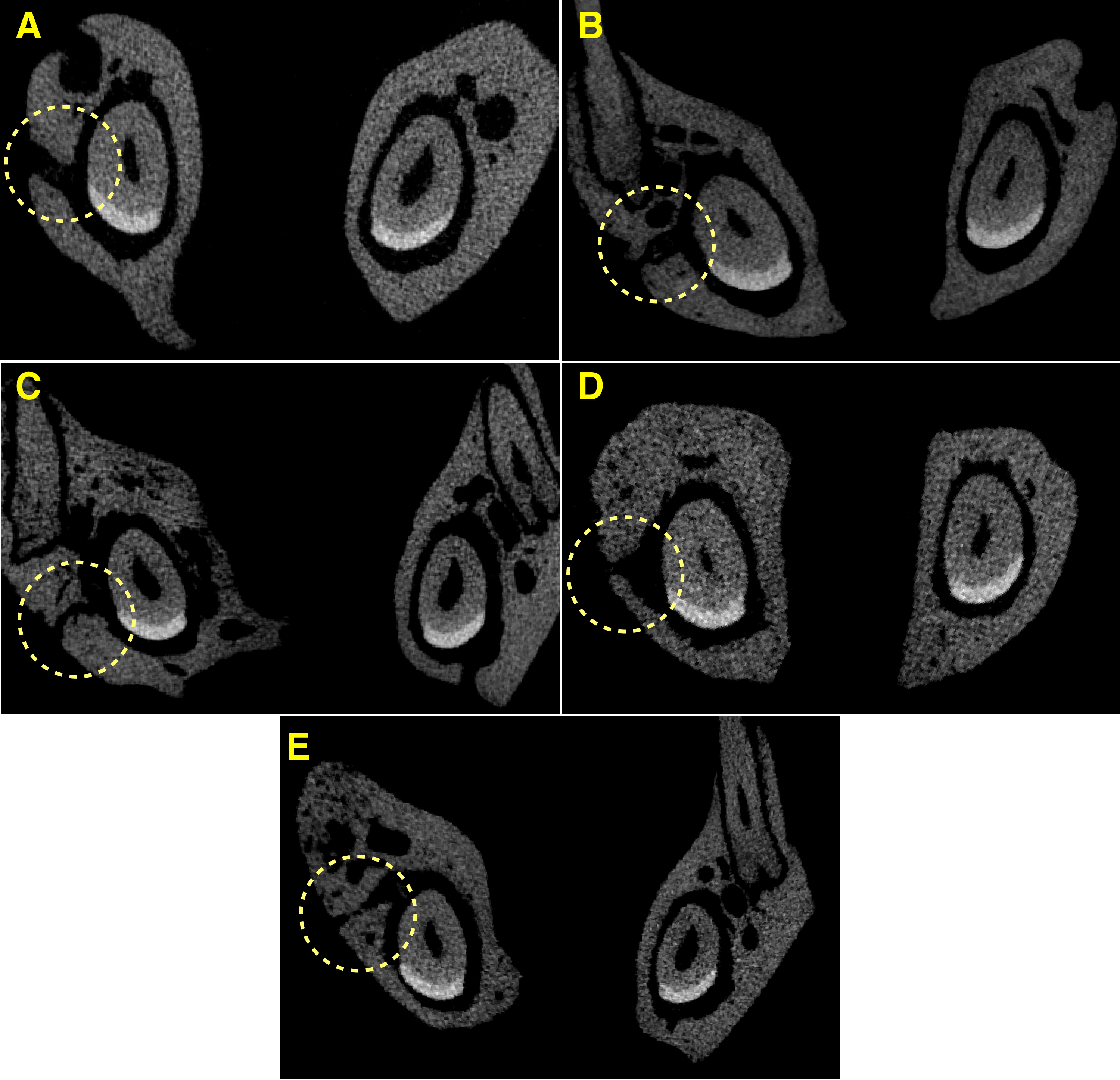


**Appendix Figure 4-** **Healing of non-critical defects by the incisor tooth in the test (ablation) group (frontal sections).** No healing was observed in the incisor defects of the five animals in the test group (n=5).


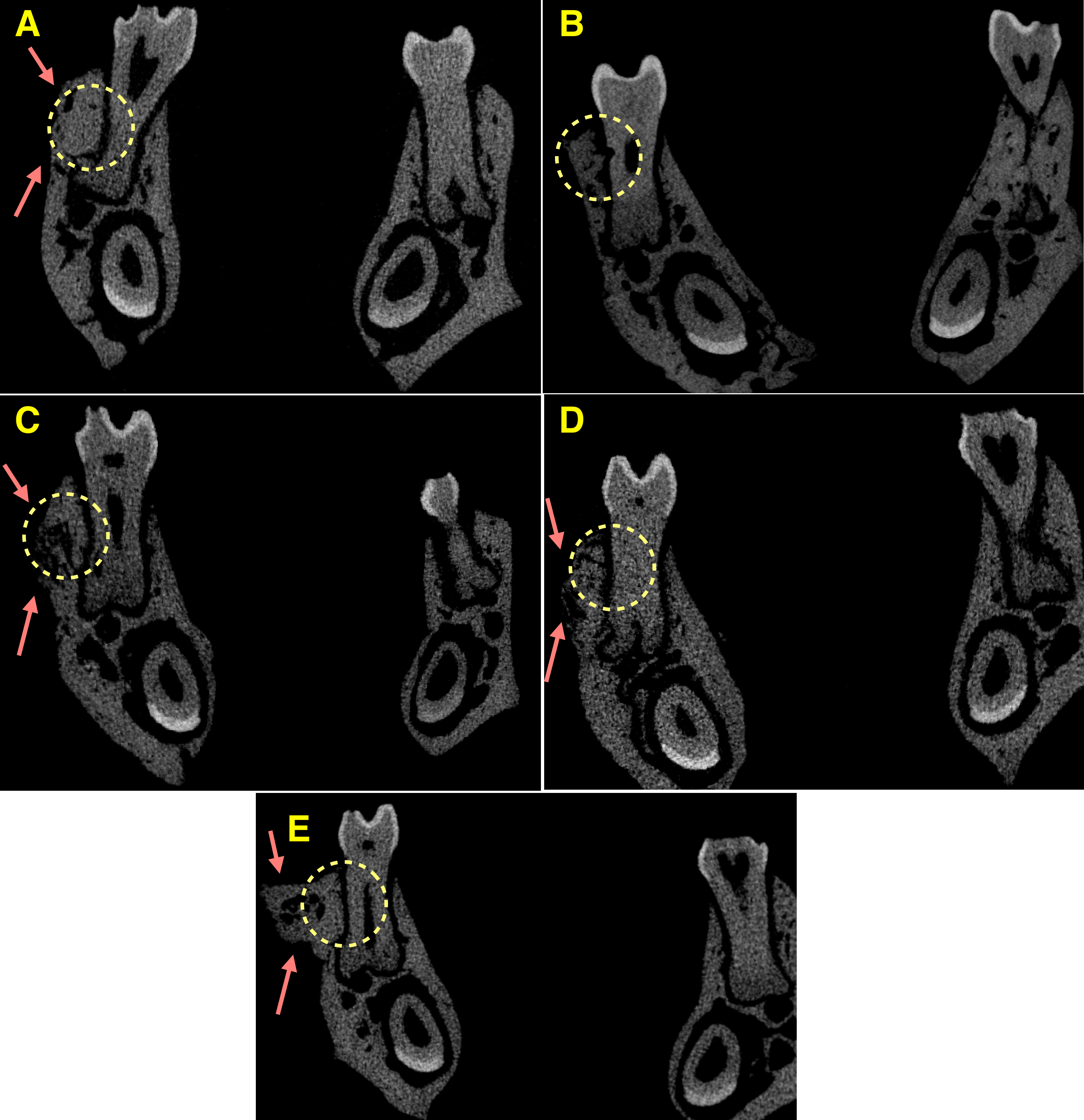


**Appendix Figure 5- Healing of non-critical defects by the molar tooth in the test (ablation) group (frontal sections).** Complete healing was observed in three defects (A, B, E), and the partial healing was occurred in two defects (C and D); healing was accompanied by excessive bone formation in four animals (A, C, D, and E; red arrows) (n=5).
